# Annotations

**Published:** 1904-07-02

**Authors:** 


### ANNOTATIONS.

### The Birthday Honours.

THE medical profession is not, it must be allowed, very prominently represented in the list of distinctions conferred in honour of His Majesty's birthday. But there need be no hesitation in acknowledging that those selected for recognition are eminently worthy of their elevation, and their appointments to honourable distinctions will be cordially endorsed by the profession. Sir Thomas Stevenson has, through a long career of highly responsible work, earned for himself a reputation for efficiency and eminence as a toxicologist and medical jurist second to none. Considering the immediate bearing of his work upon the course of public justice, it is eminently suitable that his position and services should receive the tribute of honourable and official recognition. It is in a very different field of labour that Sir Constantine Holman has applied his judgment and energies to the promotion of large and important interests. Probably his name is hardly known to any large section of the general public, and it may be that many members of his own profession are hardly aware of the valued results he has achieved. But, apart from a long and honourable career as a practitioner of medicine, he has exercised a considerable influence on the organisation and policy of the British Medical Association, and, above all, on the success and development of Epsom College. It is not too much to say that the present position of this last-mentioned institution is due to his tactful and business-like management. There is no need to dilate on the scientific work of Sir James Dewar; for though perhaps it has no very direct bearing on medical science, it is well known to all interested in modern physical and chemical developments. We may also in these columns venture to add a word of congratulation to Sir Robert Finlay, K.C., for though at an early stage of his career he deserted medicine for the practice of the law, his public services and achievements naturally have a special interest for members of the medical profession.

### Beri-Beri in the Transvaal.

FEW things could be more amusing, to a person of cynical disposition, than to observe the contrast between the ordinary indifference of all parties in Parliament to the gravest questions affecting the public health in England, and the extreme solicitude of the Opposition with reference to a reported appearance of beri-beri among the Chinese labourers recently introduced into the Transvaal. As far as we could discover from the reports of two debates upon the question, there was not a single member of either House who had the smallest conception of the nature of the disease, or who could utter an intelligible sentence with regard to it, for if exception should be made in favour of the medical members of the Opposition, they either were absent, or else they kept silence even from good words, lest a breath of common sense should exhibit the oratory of their party in its true light. Judging from this oratory alone, it might have been supposed that the disease with the unintelligible name was a sort of pestilence by which, once it was introduced, the whole of South Africa was likely to be ravaged, and it was fairly manifest that even speakers on behalf of the Government were travelling cautiously around a question of which they were almost entirely ignorant and on which they were fearful of committing themselves. A noble lord on the side of the Opposition distinguished himself by giving utterance to his belief that the emergency was far too serious a one to be left to the unaided efforts of the local authorities. In the presence of so much ignorance where knowledge might reasonably have been expected, it is permissible to say that beri-beri is a form of neuritis, attacking sometimes one set of nerves and sometimes another, and therefore giving rise to a great diversity of symptoms. As far as can be ascertained, it is dependent upon some kind of poisoning through the medium of food. It is sometimes fatal; but it is not contagious and tends towards spontaneous recovery as soon as the patients are removed from the operation of the causes. The anxieties of his Majesty's Opposition may therefore be set at rest, and some new excuse for wasting the time of Parliament will have to be discovered.

### Pharmacopœial Doses.

THE new edition of the United States Pharmacopœia, which is to be issued in the course of the next few months, is, amongst other changes, to adopt one feature of British practice, namely, the introduction of official doses for all medicines intended for internal administration. There has, it appears, been some difference of opinion on this point between the physicians on the one hand and the pharmacists on the other who are jointly responsible for the compilation of the Pharmacopœia. The latter have successfully urged the plea that in the absence of pharmacopœial doses they have no standard by which to check the doses ordered in prescriptions, and that as a consequence they are made to bear an unfair responsibility, and are not in a position to deal with any quantitative error that may accidentally be made by the physician. There must be, we think, in the medical profession a good deal of sympathy with those who oppose the definition of official doses. They are objectionable on many grounds. In the first place they appear to limit the freedom of the physician by an official definition and rule. They also suggest that the Pharmacopœia is to take a leading part in the therapeutic education of the profession with which it has essentially only the slightest possible concern. And again, official doses inevitably become quoted in text-books; they thus form a rule for the education and examination of students and are in this way only too apt to acquire an importance out of proportion to their merits and to be continued without modification as a guide for the practitioner. All this opposes wise and successful therapeutics which demands judgment and freedom in the adaptation of doses to the wants of the individual patient. Nevertheless, we agree that a national Pharmacopœia ought to quote official doses because such an arrangement forms an additional protection for the public against the risk of oversight and error and the serious possibilities attached to these; and it is for the public interest that the Pharmacopœia exists. This is a commanding consideration which cannot be ignored. The way to prevent the evil results of an official dosage is not to condemn it but to teach its true meaning and value, and thus to present it in the subordinate position it properly occupies.

